# The Role of Dendritic Cells in Graft-Versus-Tumor Effect

**DOI:** 10.3389/fimmu.2014.00066

**Published:** 2014-02-21

**Authors:** Tomomi Toubai, Nathan Mathewson, Pavan Reddy

**Affiliations:** ^1^Blood and Marrow Transplantation Program, Division of Hematology and Oncology, Department of Internal Medicine, University of Michigan Comprehensive Cancer Center, Ann Arbor, MI, USA

**Keywords:** allogeneic hematopoietic stem cell transplantation, graft-versus-tumor effect, dendritic cells

## Abstract

Dendritic cells (DCs) are the most potent antigen presenting cells. DCs play a pivotal role in determining the character and magnitude of immune responses to tumors. Host and donor hematopoietic-derived DCs play a critical role in the development of graft-versus-host disease (GVHD) following allogeneic hematopoietic cell transplantation. GVHD is tightly linked with the graft-versus-tumor (GVT) effect. Although both host and donor DCs are important regulators of GVHD, the role of DCs in GVT is poorly understood. GVT is caused by donor T cells that attack recipient tumor cells. The donor T cells recognize alloantigens, and tumor specific antigens (TSAs) are mediating GVHD. The process of presentation of these antigens, especially TSAs remains unknown. Recent data suggested that DC may be essential role for inducing GVT. The mechanisms that DCs possess may include direct presentation, cross-presentation, cross-dressing. The role they play in GVT will be reviewed.

## Introduction

Allogeneic hematopoietic cell transplantation (allo-HCT) has become widely used as a curative therapy for a variety of life-threatening hematologic, immunologic, and genetic diseases. However, serious complications endure, presenting as obstacles to successful treatment. One complication is graft-versus-host disease (GVHD) and another is primary disease relapse. The current understanding of the science suggests that the dysregulation and/or dysfunction of the immune system and corresponding immunocompetent cells of recipients after allo-HCT are responsible for these obstacles (1). Current prophylaxis and treatment regimens using immunosuppressants mainly target T cells for the mitigation of GVHD. Excessive immunosuppression for the treatment of GVHD often results in serious infections (*Cytomegalovirus*, Herpes zoster virus, fungus, and bacterias), decreases graft-versus-tumor (GVT) responses (which are the most beneficial effects of allo-HCT), and finally is known to cause relapse of primary disease (1, 2). Thus, it is imperative that we develop new strategies of GVHD prophylaxis and treatment while maintaining sufficient GVT effect.

Dendritic cells (DCs), the most potent of the antigen presenting cells (APCs) of both the innate and adaptive immune responses, are critical for the pathophysiology of both GVHD and GVL (1–3). Host and donor hematopoietic-derived APCs (particularly DCs) are critical in the development of GVHD (4–6). In addition, host hematopoietic-derived DCs also play a significant role in GVL (7, 8). In this review, we focus on the role of DCs in GVT and consider strategies for effective utilization in enhancing GVT.

## Subsets and Function of Dendritic Cells

Dendritic cells have bilateral characteristics, as DCs are critical for priming T cell responses in an inflammatory milieu, but are also required for the induction of tolerance at steady state.

Dendritic cells are phenotypically classified under many subtypes. This heterogeneity suggests that better understanding of these distinct subsets may lead to the ability to modify and manipulate DC functions. Lymphoid and non-lymphoid tissues, as well as the blood, contain a variety of DC subsets with a wide range of functions. DCs arise from bone marrow (BM)-derived macrophage/DC precursors (MDPs) (9). MDPs differentiate into monocytes, yielding macrophages; common DC precursors (CDPs), which generate classic DC (cDC)-restricted precursors (pre-cDCs); or plasmacytoid DCs (pDCs) (9). However, human equivalents of mouse MDPs and CDPs remain elusive (10). Pre-cDCs migrate from the BM and enter blood circulation destined for lymphoid organs and/or peripheral tissues. Upon arrival, pre-DCs differentiate into lymphoid/non-lymphoid tissue DCs (9). DCs express both the hematopoietic marker CD45 and integrin CD11c. Further, DCs can be divided into two major categories in lymphoid tissues, based upon the intensity of CD11c expression. The first is conventional DCs (cDCs–CD11c^high^) and second is pDCs (pDCs–CD11c^low/int^). cDCs are further categorized into lymphoid organ resident DCs and migratory tissue DCs. Both categories of cDCs are also divided into CD8α^+^DCs (lymphoid-derived DCs) and CD8^+^α^−^DCs (myeloid-derived DCs) and they show low co-stimulatory molecules in steady state (11–13). In non-lymphoid tissue, there are three types of DCs [tissue-resident steady state DCs, pDCs, and monocyte-derived DCs (moDCs)] in mouse; humans express at least two types of DCs, pDCs, and myeloid-derived DCs that are divided into three different categories: CD16^+^DCs, BDCA1^+^, and BDCA3^+^ DCs. Although DCs expressing certain phenotypes are known to contribute to development of GVHD, but not obligatory (14–16), the function of the remaining phenotypes is less understood. The various subsets are discussed very briefly below and summarized in Table 1, in light of several recent excellent reviews on these subsets (13, 17–19).

**Table 1:** **Dendritic cell subsets**.

DC subsets	Surface markers	Transcription factors	Function
Mouse: CD8α^+^DCs	Mouse: CD8α^+^ (11)	FMS-related tyrosine kinase 3 (Flt3) (171, 172)	Engulf and process exogenous antigens and subsequently present these antigens to CD4^+^ T cells via MHC class II (13) Strong cross-presentation capacity (37) Enhancement of CTL responses (38, 39)
		Interferon regulatory factor 8 (IRF8) (24, 30)	
Human: BDCA3^+^DCs	Human: BDCA3^+^ (CD141)^+^ (48)	Inhibitor of DNA binding protein 2 (Id2) (31, 36)	
	Mouse/human: MHC class II^+^, CD24^+^ (12), CD36^+^ (24), DEC205 (CD205)^+^ (12), Clec9A (DNGR-1)^+^ (22), TLR3^+^ (23), XCR^+^ (25, 46, 47)	Basic leucine zipper transcription factor ATF-like 3 (Batf3) (32)	
		Nuclear factor interleukin-3 regulated (Nfil3) (33) PU.1 (34) Zinc finger transcription factor (Zbtb46) (35)	
			Secrete large amounts of IL-12 (38, 39)
			Secretion of type I IFN with TLR3, TLR9, and plasmodium stimulation (173, 174)
			Immune modulatory function (13)
			Decrease allogeneic T cell proliferation (28, 40, 175)
			Induce FoxP3^+^ Treg and IL-10 secreting T cells (40, 41)
			Induction of peripheral self-tolerance (176)
CD8α^−^DCs	Mouse: CD8α^−^ (17), CD11b^+^ (17), CD209 (DC-SIGN)^+^ (51), CD172a (Sirpα)^+^ (52), DC inhibitory receptor 2 (DCIR2)^+^ (53), dectin-1 (Clec-7a)^+^ (54)	FMS-related tyrosine kinase 3 (Flt3) (17), lymphotoxin β receptor (LTβR) (17), notch RPB-J (55), notch receptor 2 (57), reticuloendotheliosis homolog B (RelB) (177), TNF-associated factor 6 (TRAF6) (178)	Enhancement of Th2 responses in primary stimulation (58)
			IL-12 production under certain conditions (59)
			CD4^+^ T cell activation (53)
			Cross-presentation of particular antigens under certain conditions (54, 179, 180)
Plasmacytoid DCs (pDCs)	Mouse: CD11cnt^int^ (18), B220 (CD45RA)i^hi^ (18), sialic acid-binding immunoglobulin-like lectins-H (Siglec-H)i^hi^ (18), CD317 (mPDCA-1)i^hi^ (18)	Ikaros (68), STAT-3 (68, 181), STAT-5 (181) (182)	Secretion of type I IFNs (18, 62) Immunomodulation (18) Increased cross-presentation capacity (183)
	Human: BDCA-2^+^ (60), BDCA-4^+^ (60), DCIR^+^ (61), Ly6C^+^ (62), DC-SIGN^+^ (63), CD123^+^ (64)		
Monocyte-derived DCs	Mouse (19): MHC class II^+^, CD11b^+^, CD11c^+^, F4/80^+^, Ly6C^+^, CD64^+^, M-CSFR^+^, ZBTB46^+^	Unknown	Migration into the site of inflammation from BM in a CCR2-dependent manner (77) Activation and proliferation of T cells (185– 188) Production of various cytokines (185–188)
Inflammatory DCs (infDCs)	Human (184): HLADR^+^, CD11c^+^, BDCA1^+^, CD1a^+^, FcεRI^+^, CD206^+^, CD14^+^, M-CSFR^+^, ZBTB46^+^		
Human: BDCA1DC (CD1c^+^DCs)	BDCA1^+^ (60), CD11c^+^ (79), HLADR^+^ (79), CD86^+^ (83), CCR5^+^ (83), FcγR^+^ (161)	Unknown	Secretion of high levels of IL-12, following TLR4 and TLR7 stimulation (83, 161)
			Stimulation of allogeneic T cells (79)
			Increased cross-presentation capacity (46–48, 83–85)

## CD8α^+^DCs (Mouse) and BDCA3^+^DCs (Human)

CD8α^+^DCs are approximately 20–40% of total mouse splenic DCs and around 70% of murine thymic DCs (11, 12). In steady state, they express low levels of co-stimulatory molecules, such as CD80, CD86, and CD40 but high levels of MHC class II (20, 21) and highly express CD24, CD36, DEC205 (CD205), Clec9A (DNGR-1), TLR3, and XCR, but show little or no expression of CD172a (Sirpα), CD11b, and DCIR2 (33D1) (12, 22– 26). The administration of Flt-3L to WT mice dramatically expands CD8α^+^DCs that are phenotypically and functionally matured (27) and have a reduced capacity for allogeneic T cell stimulation (28).

Certain transcription factors play an important role in the development of CD8α^+^DCs (29–36). Interferon regulatory factor 8 (IRF8) (29, 30), inhibitor of DNA binding protein 2 (Id2) (31, 36), the basic leucine zipper transcription factor ATF-like 3 (Batf3) (32), nuclear factor interleukin-3 regulated (Nfil3) (33), PU.1 (34), and zinc finger transcription factor zbtb46 (35) are critical for the development of CD8α^+^DCs. Mice lacking these transcription factors exhibit dramatically reduced numbers of CD8α^+^DCs while absence of zbtb46, which results in increased CD8α^+^DCs.

CD8α^+^DCs are unique in which they can present exogenous antigens on their MHC class I molecules, a process known as cross-presentation (37). In addition, CD8α^+^DCs are critical for cytotoxic T cell (CTL) responses as they are the predominant producers of IL-12 (38, 39). On the other hand host-derived CD8α^+^DCs, expanded by the administration of Flt-3L, decrease allogeneic T cell responses *in vivo* (28). We have also found that immunization of donors with host-derived CD8α^+^DCs, reduced acute GVHD by increased secretion of IL-10 from donor-derived T cells (40). CD8α^+^DCs can also induce Foxp3^+^ regulatory T cells (Tregs) in a TGF-β-dependent manner *in vitro* and *in vivo* (41). Moreover, CD8α^+^DCs are responsible for induction of peripheral self-tolerance by their ability to capture and cross-present tissue-associated antigens to naïve CTLs (42–44) or by CD8α^+^DCs derived TNF-mediated killing (45).

Although CD8α^+^DCs present only in mice, recent studies have identified human equivalents. BDCA3^+^ (CD141^+^) DCs, which express Clec9A and XCR-1 were identified as human homologs of mouse CD8α^+^DCs (46–49). BDCA3^+^ DCs have the ability to cross-present soluble or cell-associate antigen to CD8^+^ T cells (47, 48). Aside from the capacity for cross-presentation, BDCA3^+^DCs produce IFN-α after TLR3 stimulation, similar to CD8α^+^DCs homologs in mouse (50).

## CD8α^−^DCs (CD11b^+^DCs)

CD8α^−^DCs (CD11b^+^DCs) lack expression of the marker CD8α but express CD11b, which represent a large percentage of splenic or lymphoid resident DCs (17). CD8α^−^DCs predominately express CD209 (DC-SIGN) (51), CD172a (Sirpα) (52), DC inhibitory receptor 2 (DCIR2) (53), and dectin-1 (Clec-7a) (54). Notch RBP-J, is important for development and homeostasis of CD8α^−^DCs (55). Recent reports also suggest that Notch 2 signaling is required for the development of a subset of splenic CD11b^+^ DCs (CD11b^+^ESAM^+^DCs) and intestinal CD103^+^CD11b^+^DCs (56), as well as terminal differentiation of CD8α^+^DCs and CD11b^+^DCs (57). CD8α^−^DCs are required to enhance Th2 responses in primary stimulation (58) and also they produce IL-12 under certain conditions (59). CD8α^−^DCs exist in the marginal zone of the splenic lymphoid follicles and take up, process, and present exogenous antigen to CD4^+^ T cells via MHC class II (17, 53).

## Plasmacytoid DCs

Plasmacytoid DCs are distinguished in mice by the expression of CD11c^int^, B220 (CD45RA)^hi^, sialic acid-binding immunoglobulin-like lectins-H (Siglec-H)^hi^, and CD317 (mPDCA-1)^hi^ (18). In human, pDCs express BDCA-2 (60), BDCA-4 (60), DCIR (61), Ly6C (62), DC-SIGN (63), or CD123 (64). Flt3-L is a critical cytokine for the expansion of pDCs (65, 66), whereas HIF-1α is a negative regulator of pDC development *in vitro* and *in vivo* (67). Ikaros and STAT-3 play a role in the development of pDCs (68). The main function of pDCs is to produce type I interferons (IFN), such as IFN-α and IFN-β, in response to viral, fungal, and bacterial antigens (18). The role of pDCs in mediating acute GVHD is distinct depending on whether they are derived from the host or donor (69, 70).

## Monocyte-Derived DCs

According to recent reports, monocytes exist in the blood as terminally differentiated cells derived from MDP [whose progenitor is common myeloid precursors (CMPs) in the BM]. In an inflammatory environment, monocytes differentiate into MoDCs, or inflammatory DCs (infDCs) and subsequently migrate into the site of inflammation (71, 72). Monocytes also contribute to the development of CD103^−^CD11b^+^DCs in a Csf-1-dependent manner (73, 74). Mouse BM-derived DCs generated *in vitro* with GM-CSF alone or in combination with IL-4 are recognized as equivalent to infDCs because of similar morphology, phenotype, and characteristics (75, 76). CCR2 controls the exit of monocytes from the BM and the migration to the site of inflammation and critical for infDCs. Further, MyD88 and TLRs are known to be required for the maturation and migration of infDCs (77, 78).

## Human BDCA1 (CD1c)^+^ DCs

Dendritic cells isolated from human are identified as Lin^−^ (CD3, CD19, CD14, CD20, CD15, glycophorin A) CD11c^+^HLADR^+^ cells (79) and are classified into three groups based on their expression of BDCA1, BDCA3, and CD16 (60). BDCA1^+^ (CD1c^+^) DCs are one of the blood DC subsets found, in addition to lymphoid tissue-resident DCs and those observed in the skin of humans (79–81). BACA-1^+^DCs are likely the human counterpart of murine CD11b^+^DCs (82). BDCA1^+^DCs have a strong capacity for allostimulation (79) and can cross-present exogenous antigen to CD8^+^ T cells but less efficiently than BDCA3^+^DCs (46–48, 83–85).

## DC Chimerism after Human Allogeneic HCT

Although the replenishment of recipient DCs depends on donor hematopoietic stem cells (HSCs) and associated precursors, the exact half-life of host APCs in especially inflamed tissues is not well-understood. So far, kinetics of DC engraftment and turnover (DC chimerism) utilizing myeloid specific or directly staining DCs in peripheral blood mononuclear cells (PBMCs) after allo-HCT, have been reported in humans (86–99). Most of these reports demonstrated that the reconstitution of human DCs (myeloid CD11c^+^DCs and plasmacytoid CD123^+^DCs) in the early phase of allo-HCT show that nearly complete donor-derived chimerism (CDC) develops and maintains in the late phase. However, a small population of recipient-derived DCs may exist long-term (86, 90, 93, 94). Interestingly, patients with acute GVHD showed significantly lower donor chimerism of DCs as well as low numbers of circulating DCs (93, 94, 96). Further, 6-sulfo lac NAc DCs (slan DCs), potent producers of inflammatory cytokines following LPS stimulation (100) are a major subpopulation of human blood DCs and are also reduced in the patients with severe acute GVHD (92). Although it is helpful to examine the kinetics and chimerism of the peripheral circulating DCs, the kinetics and activation of tissue-resident DC subsets in recipient (especially GVHD-associated organs and/or lymph nodes) might play a role in the development of GVHD.

Host-derived Langerhans cells (LCs) are rapidly depleted by myeloablative regimens and are quickly replaced by donor type in the absence of GVHD. The recovery of donor LC chimerism and numbers, however, are delayed in the presence of acute GVHD (98, 99). In the skin, host-derived myeloid DCs (such as CD1a^+^ and CD14^+^DCs) are quickly replaced by donor cells, where host-derived macrophages still exist during GVHD (97). Similar to the relationship between GVHD and DC kinetics, a decrease in number of DCs is observed (96) and mixed chimerism in DCs has the capacity for a potent GVT effect in donor lymphocyte infusion (DLI) (101). This suggests a positive impact of host-derived DCs on GVT effect.

## DCs after Experimental Allogeneic HCT

The results from experimental allo-HCT suggest a complicate role for DCs in GVHD. For instance, cDCs and pDCs are activated by TBI (102) and inflammatory cytokines (103, 104) (IL-1 and TNF-α), which are released by damaged tissues. These activation signals up-regulate the expression of antigen presenting and co-stimulatory molecules and could modulate GVHD (102). Moreover, when all other hematopoietic APCs are absent, DCs alone may induce GVHD (5, 105). However, recent reports indicate that host-derived hematopoietic APCs are dispensable for inducing GVHD, specifically CD11c^+^DCs and/or pDCs depletion in the presence of other APCs (106, 107) does not attenuate GVHD, it might even increase lethal GVHD (15, 107). These data clearly demonstrate that host DCs are therefore not crucial for the induction of GVHD and could even play a regulatory role. On the other hand, donor-derived APCs, especially cDCs too are not required for induction of GVHD, but may play a role in maintenance or aggravation of GVHD in presence of other hematopoietic APCs (6, 106).

## DCs and GVT

**Figure 1:**
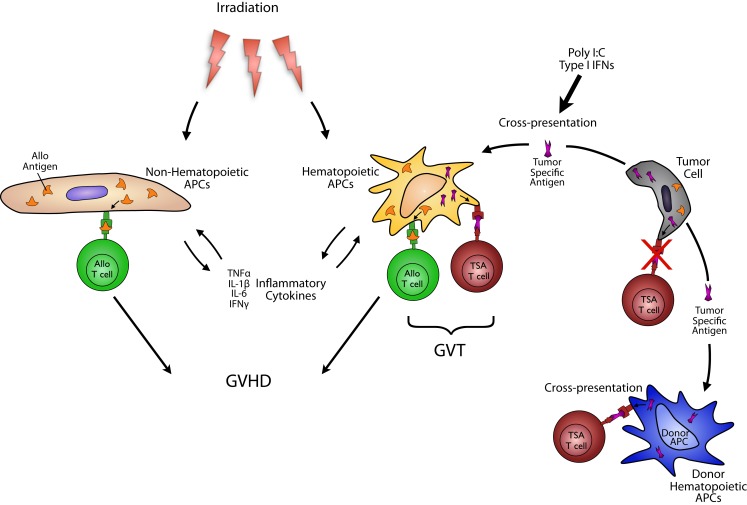
**Antigen presenting cells in GVT: to maximize GVT responses, two important factors must be considered: antigen presentation and donor T cells**. Although both host and donor DCs have been shown to play an important role in GVHD, their role in GVT is only beginning to be understood. To induce GVT, although donor T cells must recognize both alloantigens and TSAs that presented either directly by the tumor or indirectly by the professional APCs, tumor themselves are generally poor presenters and activators of T cell effector responses. Therefore, professional APCs are required for optimizing GVT. While alloantigen responses are also elicited by many APCs including both hematopoietic-derived and non-hematopoietic-derived APCs cause GVHD, TSAs are exclusively directed to tumors and thus considered to GVHD without concomitantly causing GVHD. In cases where tumors are poor APCs of TSAs to donor T cells, the TSAs have to be efficiently presented by professional APCs, especially DCs, derived from either donor or host. This mechanism of presentation includes cross-presentation.

To maximize GVT responses, two important factors must be considered: antigen presentation and donor T cells. Although both host and donor APCs have been shown to play an important role in GVHD, their role in GVT is only beginning to be understood. Donor T cells have to attack recipient tumor cells in GVT. To that end, they must recognize both alloantigens and tumor specific antigens (TSAs) that presented either directly by the tumor or indirectly by the professional APCs (Figure 1). There is a large amount of evidence that tumor themselves are generally poor presenters and activators of T cell effector responses. In the context of allo-HCT, professional APCs are required for GVT. Their requirement, however, when certain leukemia or tumors may efficiently present antigens to donor T cells have not been obviously analyzed. Nonetheless, GVT responses are optimal when both alloantigens and TSAs responses are induced (7). While alloantigen responses are also elicited by many APCs including both hematopoietic-derived and non-hematopoietic-derived APCs cause GVHD, TSAs are exclusively directed to tumors and thus considered to GVT without concomitantly causing GVHD. In cases where tumors are poor APCs of TSAs to donor T cells, the TSAs likely have to be efficiently taken up and cross-presented on professional APCs. In this regard, DCs may be most relevant and could employ three possible mechanisms they possess better than other hematopoietic APCs, capability for better cross-presentation and cross-dressing.

Clinically, most patients with allo-HCT receive HSCs and T cells from human leukocyte antigen (HLA) matched, but multiple minor histocompatibility antigens (MiHAs) mismatched donors. This difference in MiHAs between host and donor are targets for donor T cells to mediate GVH responses. Alloantigen is expressed by all host APC subsets as endogenous antigen, which they directly present to donor CD8^+^T cells, even if the interaction is brief (4). In addition to MiHAs, donor T cells respond to TSAs that are virally encoded and/or mutated tumor antigens representing additional important targets for GVT responses. Activated and proliferated allogeneic T cells, stimulated by APCs, are “double edged swords” in that they not only attack host residual tumors but also damage normal host tissues. Augmenting GVT responses through identification of relevant TSAs and determining T cells that specifically respond to them is clinically challenging because GVHD is an allo-reactive disease enhancing TSA-specific T cell responses, which are dependent on allogeneic reactions (108, 109). As one approach to distinguish this clinical dilemma, recently, MHC class I-associated tumor-specific phosphopeptides presented on hematological tumors were shown to be critical for induction of their specific memory-like CD8^+^T cells against leukemia and that the response against leukemic patients can be restored after allo-HCT (110). These suggest that DCs must simultaneously present both alloantigens, derived primarily from the endogenously polymorphic peptides in the host target tissues, and TSA to donor CD8^+^ and CD4^+^ T cells via MHC class I and class II molecules, respectively. In the clinic, the importance of host APCs in GVL has been suggested in patients with mixed chimerism after DLI in non-myeloablative BMT (111).

We and others have experimentally explored the role of APCs in GVL. Host type APCs are required to maximize GVT responses after allo-HCT (7) and after DLI because they prime donor CTL in an effective manner (112–115). Host MHC class II^+^ APCs and CD4^+^ T cells have an indispensable role in CTL responses in mixed chimera models (112). In addition, donor T cells primed by leukemia lysate-pulsed host APCs before DLI, enhance GVT responses in either leukemia-bearing full chimera or mixed chimera models (113). These data suggest that the host environment is critical for mediating GVT responses. Host type sialoadhesin^+^ macrophages, which increase inducible nitric oxide (iNOS) production by CD40–40L interaction in the liver, stimulate CTL and prevent liver metastasis (116, 117). Based on the fact that host leukemia cells or tumors express alloantigens, in addition to TSA, may possess co-stimulatory molecules, they could be “APCs.” Although they express APC like features, they have likely undergone a process of “immune-modulating,” making them poor direct stimulators of an effective T cell response using a variety of immune-suppressive mechanisms. We have shown that certain lymphoma cells lines, despite some APC features, are not capable of driving an efficient GVT response in the absence of hematopoietic-derived APCs (7).

We have explored, more recently, the APC subsets that are required for optimal GVT without GVHD. We recently found that host-derived CD8α^+^DCs are required for the induction of optimal GVT responses utilizing Batf3 deficient mice as recipients in experimental allo-HCT (8). We also found that TLR3 stimulation via poly I:C in host CD8α^+^DCs, enhanced GVL responses without exacerbating GVHD (8). As we described previously, CD8α^+^DCs are critical for cross-presentation of tumor and viral antigens (32, 118, 119) because of their well-specialized cross-presentation capacity and their superior ability to prime antitumor CTL responses (32, 119–121) without enhancing GVHD (8, 122). As noted above, recently human BDCA3^+^, XCR-1^+^, DNGR-1^+^DCs found in spleen, blood, and non-lymphoid tissues are recognized as the equivalent of murine CD8α^+^DCs by multiple investigators (26, 46–48, 123). Therefore, our investigations underscore the principle of enhancing antigen presentation using a subset of host APCs as a strategy for effective enhancement of GVT responses following allo-HCT. However, cellular processes of regulating GVT responses in host APCs still remain unclear. Specifically whether low numbers of CD8α^+^DCs reduce TSA responses or decrease GVT responses remain unknown. We also explored the molecular mechanism in hematopoietic-derived APCs for enhancing GVHD. The absence of Ikaros in host hematopoietic APCs exacerbates GVHD, but without concomitantly enhancing GVT responses in multiple models (unpublished data). This uncoupling is an interesting phenomenon as GVT responses are usually tightly linked with GVHD severity. Furthermore, genetic alteration of Ikaros family zinc finger protein 1 (IKZF1) in acute lymphoblastic leukemia (ALL) is associated with poor outcome and high relapse after chemotherapy (124, 125). Therefore, we are pursuing whether Ikaros in leukemic cells alone or both leukemic and non-leukemic host hematopoietic cells play a role in mediating GVT resistance.

Understanding the host microenvironment, especially that of the tumor is essential for GVT studies. Tumor-infiltrating DCs in tumor microenvironments in hosts are suggested to regulate CTL responses, however, their role in the context of allogeneic HCT remain obscure.

The role of donor-derived DCs in mediating GVT is also being explored. Initial reports regarding this association demonstrated that donor APCs are not required for GVT responses, but play an indispensable role in GVHD in MHC matched, MiHA mismatched BMT model (6). In order to present host TSAs via donor APCs to donor CD8^+^T cells, donor APCs must have the capacity for cross-presentation as they do not express both endogenous alloantigens and TSAs. Furthermore, additional studies are needed to determine which specific subsets of donor APCs play a critical role in enhancing GVT responses. Reports suggest that donor CD11b^−^ APCs within the BM grafts consist mostly of pDC progenitors (pre-pDCs) and enhance GVT activity of donor T cells by promoting differentiation into Th1/type 1 CTLs. These effects have further been shown to be mediated by IL-12 in murine allo-HCT models (126, 127). Pre-pDCs also regulate GVH and GVT responses altering the balance between donor Tregs and inflammatory T cells by inducing indoleamine 2,3-dioxygenase (IDO) synthesis (128). In humans, however, there are no data of the exact mechanisms of specific subsets of donor APCs in GVT. Therefore, studies examining and elucidating the kinetics of these subsets of DCs would contribute to likely better understanding the mechanism of GVT in humans.

Recent reports suggest a paradoxical association between CMV reactivation after allo-HCT and reduced disease relapse (129–131). The mechanisms that CMV reactivation induces potent GVT are still unclear. However, donor APCs and NK cells might play an important role in this interesting phenomenon (132). Interaction between cDCs and NK cells is critical to the activation of effective antiviral or antitumor response (133, 134). It is possible that donor DC–NK cell interactions might play a role in enhancing GVT mediated by NK cells in this context.

## Cross-Presentation and GVT

Dendritic cells are well-known to take up exogenous antigens via endocytosis or phagocytosis. Antigen is then processed in the endoplasmic reticulum (ER) and presented via Class I molecules. These processes are known as cross-presentation. Although the molecular mechanism of cross-presentation is still under investigation, two major intracellular pathways of cross-presentation are speculated. One is cytosolic and the other one is a vacuolar pathway (135). The cytosolic pathway depends on the proteasome, which degrades internalized proteins in the cytosol. The degraded peptides are then transported into the ER in a transporter associated with antigen processing 1 (TAP1) and TAP2-dependent manner. Peptide is then either loaded onto MHC class I molecules (ER loading) or re-imported into the phagosome to be loaded onto MHC class I molecules (phagosomal reloading) (135). A novel molecular mechanism utilizing the small GTPases Rac1 (CD8α^−^DCs) and Rac 2 (CD8α^+^DCs), regulate phagosomal oxidation, which is critical for the cross-presentation capacity (136). In addition, soluble *N*-ethylmaleimide-sensitive factor attachment protein receptor (SNARE) Sec22b plays an important role in phagosomal function through the recruitment of ER proteins into the phagosome (137) and heat shock protein 90 (HSP90) contributes to cytosolic translocation of extracellular antigen, enhancing cross-presentation (138). Conversely, the vacuolar pathway is known to be a TAP and proteasome independent pathway (139– 141) where exogenous antigens are degraded in the phagosome and subsequently loaded on MHC class I. This pathway is sensitive to cathepsin S inhibitors (140). Some DCs, such as those that express CD8α^+^ (32, 37, 142, 143), CD103^+^ (144– 147) in mice, and BDCA3^+^DCs (functional homology to mice CD8α^+^DCs) (46, 123, 148– 150) in human are known to have the capacity for cross-presentation. However, some recent reports suggested that nearly all DCs have the capacity for cross-presentation depending on the source of antigen, cytokine milieu, and expression of immunoreceptors specialized to take up exogenous antigens (76, 83, 149, 151). The role of cross-presentation in GVT responses is still unknown. Our data indicates a role for CD8α^+^DCs and also suggested that TLR3 agonist, polyI:C, can increase GVT without enhancing GVHD in host DC-dependent manner (8). Therefore, we presume that specialized DCs could be associated with optimizing GVT responses because mouse CD8α^+^DCs and human BDCA3^+^DCs possess the most potent cross-presentation capacity of TSAs. However, direct *in vivo* demonstration enhancing cross-presentation by CD8α^+^DCs or TLR3 agonist in increasing GVT has not been shown. While these are being explored, at the minimum our data suggested a novel concept that it is feasible to modulate host DCs to improve GVT without increasing toxicity. It remains to be tested, however, whether this concept holds true for all leukemia or tumors. In any event, it does suggest a window of opportunity for careful design of clinical trials in high-risk leukemia.

## Cross-Dressing and GVL

Recently, another means of antigen presentation, called “cross-dressing” was forward by Ostrand-Rosenberg’s group in 2006 (152, 153). It is postulated that cross-dressing transfers cellular materials (such as peptide MHC to DCs) triggering DC activation and enhanced tumor-specific CD4^+^ T cells in cancer vaccine (153). In 2011, as a breakthrough mechanism of elicited CTL responses by DCs, preformed peptide MHC class I complex is expressed on infected cells and can be transferred to uninfected DCs without requiring other antigen processing. This process mediates the activation of memory CD8^+^ T cells after viral infection (154). CD8α^+^CD103^+^DCs are thought to play an important role in not only cross-presentation but also cross-dressing to prime CTLs following vaccination (155). Its role suggested in GVHD but GVT responses is still unknown.

## The Strategy of Augmenting GVT Responses Utilizing DCs

Graft-versus-tumor is tightly linked with GVHD and is very difficult to uncouple the two. However, recent advances and understanding of DC biology make treatment regimens previously not considered, namely modulating antigen presentation, to now be practical options. Nonetheless much remains to be understood. Specifically, comprehensive understanding of DC subsets will enable us to maximize GVT responses. For instance, either by enhancement of cross-presentation, increased NK cell activation, or induction of type I IFN etc.

We and others have shown that administration of poly I:C stimulates TLR3 on CD8α^+^DCs enhancing cross-presentation and direct presentation to CTLs against tumors and virus infection (8, 118). In addition, poly I:C administration also activates NK cells through the enhancement of myeloid DC–NK interaction mediated through an IRF-3-toll/interleukin 1 receptor homology domain-containing adaptor molecule (TICAM-1)-IRF-3-dependent NK-activating molecule (INAM) axis-dependent manner (134). Moreover, CD8α^+^DCs treated by poly I:C can activate NK cells in the IFN-promoter stimulator-1 (IPS-1) and Toll/IL-1R domain-containing adaptor inducing IFN-β (TRIF)-dependent manner (156). Therefore, poly I:C treatment after allo-HCT could be extended to increase GVT, however, poly I:C in this context must be carefully studied as it may enhance GVHD.

Careful utilization of exogenous type I IFN (IFN-α/β) administration may also be a valuable method of enhancing GVT responses because they play an important role in cross-presentation of tumor antigens on DCs, especially CD8α^+^DCs, and enhance CTL responses (119, 120, 157). In murine allo-HCT models, exogenous type I IFN administration augments CTL responses through the increased sensitivity of host target tissues and leukemia to respond to cell mediated cytotoxicity in CD8-dependent GVHD/GVT model regardless of decreasing GVHD response in CD4-dependent model (158).

Other strategies to enhance antitumor responses through the augmentation of the cross-presentation capacity of TSA and activation of CTLs may also be feasible. Alpha-alumina nanoparticles (159), poly (γ-glutamic acid)-based nanoparticles (γ-PGA NPs) (160), Fcgamma-receptor (FcγR) antigen targeting (161), TLR7 stimulation by polyuridylic acid (polyU), which is a synthetic ssRNA analog (162), vitamin E analog-α-tocopheryl oxyacetic acid (α-TEA) (163) may be useful, but have not been studied in GVT models. Modulation of host type DCs with anti-CD3 pre-conditioning is also an efficient strategy for separating GVT and GVHD (164). Furthermore, recent modulation of DCs by reagent-based inducible or constitutive methods suggested that deep deletion of host cDCs, pDCs, and B cells are dispensable for decreased GVH responses (107). This indicated that very low numbers of DCs, or all host cells including non-hematopoietic APCs, can directly present alloantigen. Alloantigen expression on host non-hematopoietic cells decreases GVT responses in a PD-1/PD-L1-dependent manner in murine experimental BMT (165). Given this, enhancement of function in only certain DCs specialized for TSA presentation may also increase GVT responses without exacerbating GVHD. Moreover, experimental data suggested that modulation of DC function with HDAC inhibitor can result in immunomodulation to reduce GVHD (166).

Aside from enhancement of the presentation capacity in DCs, disruption of negative regulatory interactions is also important for GVT responses. PD-1/PD-L1 interactions and CD47–SIRP-α interactions are thought to be critical immunosuppressive function in the tumor environment. For instance, because the expression of PD-1 on T cells and PD-L1 on APCs facilitated increased Tregs and decreased CTL functions, PD-1/PD-L1 blockade with anti-PD-L1 monoclonal antibody decreased the infiltrating number of Tregs and increased the number and function of tumor reacting CTLs in an AML mouse model (167). Furthermore, knock down of PD-L1 and PD-L2 on MoDCs by utilizing siRNA demonstrated augmented expansion and function of MiHA-specific memory and effector CD8^+^ T cells from leukemia patients *in vitro* (168). These data suggested that anti-PD-L1 and PD-L2 blockade might be a potential strategy for the enhancement of GVT responses. Tumors may also escape from tumor surveillance utilizing the interaction between monocytic CD47 and SIRP-α, which is an inhibitory receptor of phagocytosis (169). Recent report showed engineered high affinity SIRP-α variants can disrupt this interaction and increase phagocytosis of cancer cells and enhance antitumor response (170). Although we do not know how these pathways affect GVHD, such strategy may also be considered as potential option to treat patients with high risk leukemias.

## Closing Remarks

Dendritic cells play important roles in both GVHD and GVT. Because DCs are heterogeneous, the role of specific DCs in GVHD and GVT in the presence or absence of other hematopoietic-derived APCs will need further examination. Identification of a specialized subtype of DC that may increase GVT without enhancing GVHD, such as CD8α^+^DCs in mice, may be possible. Functional studies have identified direct antigen presentation capacity, cross-presentation, and cross-priming of CTLs as critical mechanisms in allo-HCT. To enhance GVT response, both alloantigen and TSA must be presented to CTLs. However, tumor cells themselves have a poor antigen presentation capacity, therefore TSA are cross-presented by APCs. Enhancement of the cross-presentation capacity has the potential to increase GVT response and be a presumably new strategy in allo-HCT. Through the utilization of DCs, the goal of increasing GVT and diminishing GVHD might be realized.

## Conflict of Interest Statement

The authors declare that the research was conducted in the absence of any commercial or financial relationships that could be construed as a potential conflict of interest.
